# Maritime Aerosol Optical and Microphysical Properties in the South China Sea Under Multi-source Influence

**DOI:** 10.1038/s41598-019-54483-6

**Published:** 2019-11-28

**Authors:** Chi Zhang, Hua Xu, Zhengqiang Li, Yisong Xie, Donghui Li

**Affiliations:** 1 State Environmental Protection Key Laboratory of Satellite Remote Sensing, Aerospace Information Research Institute, Chinese Academy of Sciences, Beijing, 100094 China; 2 University of Chinese Academy of Sciences, Beijing, 100049 China

**Keywords:** Atmospheric dynamics, Environmental impact

## Abstract

The South China Sea hosts a wide range of aerosol pollutants with the uneven development of socio-economic and complicated meteorology system. To fill the gap of the maritime aerosol characteristics over the sea, we selected the multi-year ground-based measurements of Taiping Site and Dongsha Site to investigate the optical and microphysical properties. In Taiping, the vast majority of aerosol optical depths (AODs) are less than 0.2, but that of Dongsha shows the wider distribution of AODs from 0 to 0.6. Angstrom Exponent frequency distribution in Taiping peaks at the range of 0.75–1.25 but that has the left-skewed distribution in Taiping Island. Moreover, there is a variation in the coarse-mode volume concentration in Taiping Island but less variation in the fine mode. The seasonal maritime aerosol properties of Taiping and Dongsha have been analyzed that can be employed as a maritime look up table (LUT) kernel in coupled atmospheric retrieval and correction algorithms.

## Introduction

The South China Sea is a low-latitude marginal sea in Southeast Asia and directly located in the outflow of Mainland China and Indochina Peninsula, mixed with the pollution of the Maritime Continent^1^. The complex physical, socio-economic and biological geography and the complicated monsoon climate play a important role in the joint effect of the properties and distribution of aerosols^2,3^. Moreover, surrounding the South China Sea, there are different sources of aerosol pollution due to the various economy development conditions^4^.

Much attention has been given to this area study. Sampling method of atmospheric particulates is one major method to study the aerosol chemical properties and related pollutants in this region. See *et al*. offered  a glimpse of the particles properties in Southeast Asia compared with that in other places^5^. Chuang *et al*. found at Dongsha Island the water-soluble ions and carbonaceous content are the main components of the *PM*_2.5_ and *PM*_10_ and the resolved carbonaceous fraction were mainly distributed in *PM*_2.5_ which influenced by coal combustion, mobile vehicles and biomass burning^6^. Moreover, remote sensing provides a fundamental tool to understand the role of aerosols in the climate and earth system. The aerosol properties can be achieved by the space- and ground-based measurements. Wang *et al*. analyzed the Lidar data and found the strong Asian dust storm effected large areas from the Gobi desert to the West Pacific^7^. The similar results were also reported in many studies^8,9^. Smirnov *et al*. has analyzed the global maritime aerosol properties in a large-scale method by the ground-based data of AERONET and ship-borne measurements^10–12^. Many researchers investigated the local aerosol characteristics and analyzed the hygroscopic growth, radiative properties and pollutant sources based on the ground-based measurements surrounding the South China Sea^13–16^.

Although the above-mentioned researches focused on the aerosol properties and analyzed the mechanism of the local pollution, they only employed the short-time measurements during an experiment, or mainly concentrated on the high aerosol load cases. So it still remains a big challenge to obtain the overall optical and microphysical properties in the South China Sea, which is important to study the pollution source and climate change in this region, and fill in the gap of aerosol models over the area.

In this paper, we employed the ground-based measurements to research on the maritime aerosol properties in the South China Sea that the two sites are located in the north (Dongsha) and south side (Taiping) of the sea. Based on the multi-year data, the complexities of source, meteorology and geography are taken into account to summarize the seasonal maritime aerosol models in the South China Sea, which play a role in the research on regional climate as well as the algorithms of satellite retrieval and the atmospheric correction of satellite images.

## Data

The CIMEL Electronique CE-318 sun-sky radiometer is a part of the AERONET (https://aeronet.gsfc.nasa.gov/, Data last accessed on 29^th^ Apr 2019) and the details are described^17^. The CE-318 has wavelengths of 340, 380, 500, 675, 870 and 1020 nm (1640nm in the extended wavelength Cimel versions) for aerosol measurements and 936 for perceptible water. The optical, physical and radiative properties of the total columnar aerosols can be obtained from the direct and diffused sun light measurements used in the standard inversion algorithm of AERONET^18,19^. There are three levels of AERONET products. Briefly, Level 1.0 refers to the raw data calculated from measurements and calibration coefficients and instrumental corrections. Level 1.5 is based on the data of Level 1.0 but screening cloud by the automatic procedures^20^. Level 2.0 has the additional application of pre- and postal calibration coefficients and expert checking.

**Figure 1:**
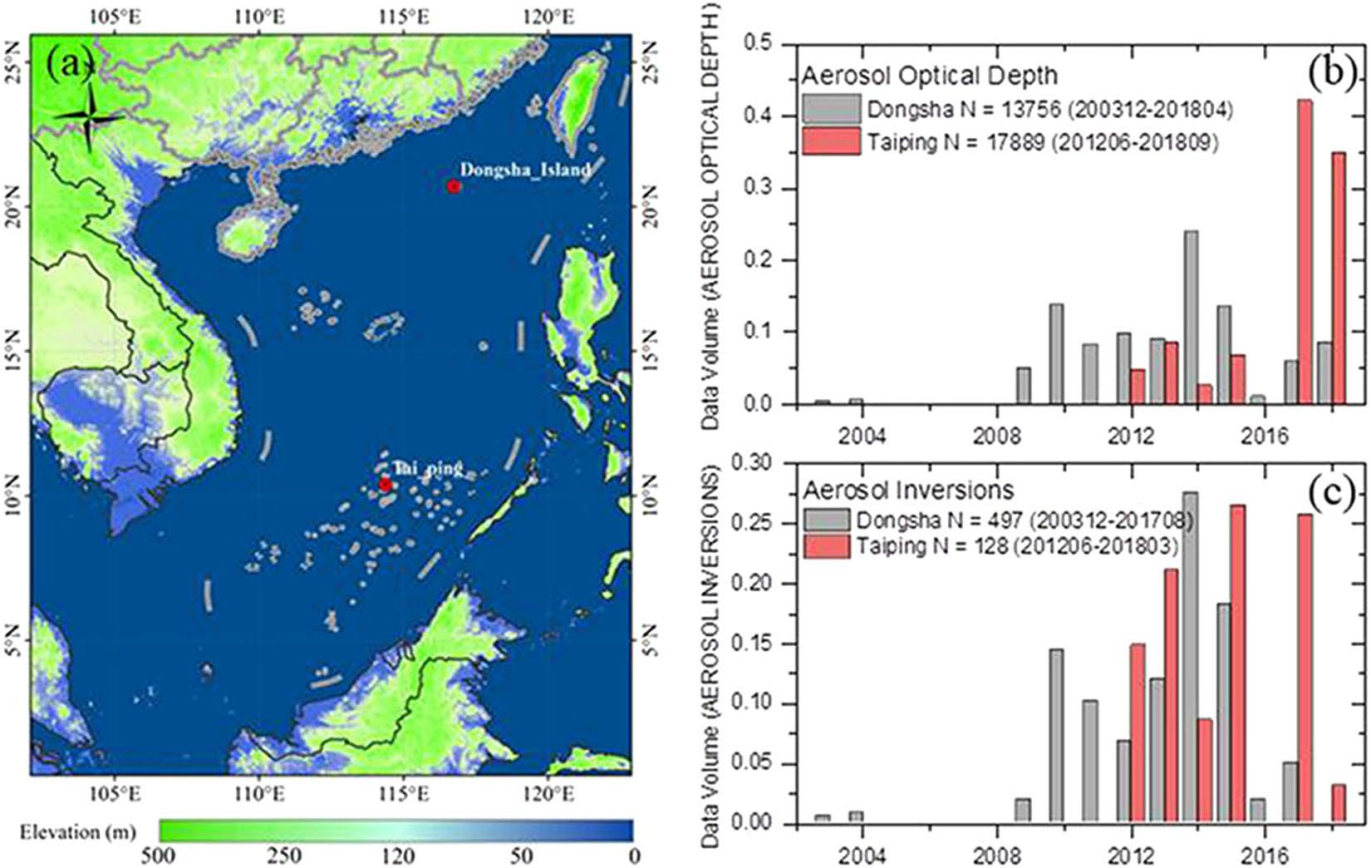
Map of the South China Sea and the location of ground-based sites (**a**) and the annual Level 2.0 data volume distribution of aerosol optical depth (**b**) and aerosol inversions (**c**) over each site.

In this study, we chose 2 ground-based sites in the island of Taiping and Dongsha over the South China Sea from AERONET. Figure 1 presents the location of each site. Dongsha Island is located in the northern region and Taiping is the southern site in the South China Sea. The data volume in Level 2.0 of each site is shown in the histogram graphs in Fig. 1. The upper one displays the aerosol optical depth (AOD)-related products calculated from the direct-sun measurements and the below one shows the inversion products from the sky radiance measurements in the almucantar geometry. Although the data of Dongsha are obtained from 2003 which is long before that of Taiping, the most of data in two sites concentrated over the last decade. As for the site of Dongsha, there are 497 pieces (239 days) of inversion data and 13756 pieces (815 days) of AOD-related data. And in Taiping, there are 128 pieces (79 days) of inversion data and 17889 pieces (506 days) of AOD-related data.

## Analysis

### Optical properties

**Figure 2:**
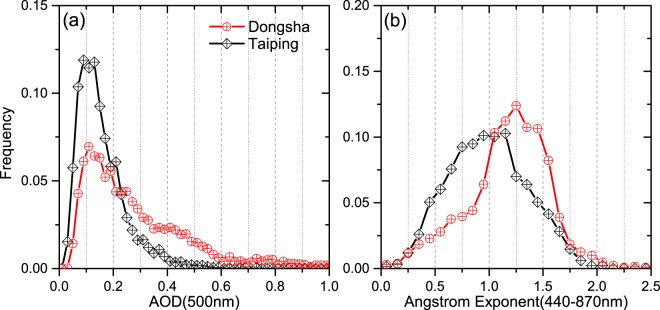
Frequency of occurrences of daily-average aerosol optical depth at 500 nm (**a**) and Angstrom Exponent (440–870 nm) (**b**) of Taiping and Dongsha site.

There is a significant amount of data accumulated in site of Taiping and Dongsha of AERONET. Figure 2(a) presents the frequency of occurrence distribution for the daily-average AOD at 500 nm and Angstrom Exponent (AE, 440–870 nm) of Level 2.0. The frequency distribution for Taiping shows that the vast majority of AODs are less than 0.2 and the mode is situated at 0.1, but that of Dongsha shows the wider distribution of AODs from 0 to 0.6. Compared with the results in Pacific, Atlantic and Indian Ocean^10^, the AOD distributions of the sites in the South China Sea are broader. The largest AODs (500 nm) in the sites of Taiping and Dongsha are 0.78 and 2.03, respectively.

In Fig. 2(b), the AE parameter frequency distribution in Taiping has a maximum at 2.12, peaks at the range of 0.75–1.25. However, in Dongsha, the peak of frequency distribution is shewed towards right which indicates more data in the range of AE more than 1. Compared to the results of Smirnov *et al*.^10^, the parameter of AE in the two sites focuses on the higher values indicating more fine-mode particles.

### Microphysical properties

**Figure 3:**
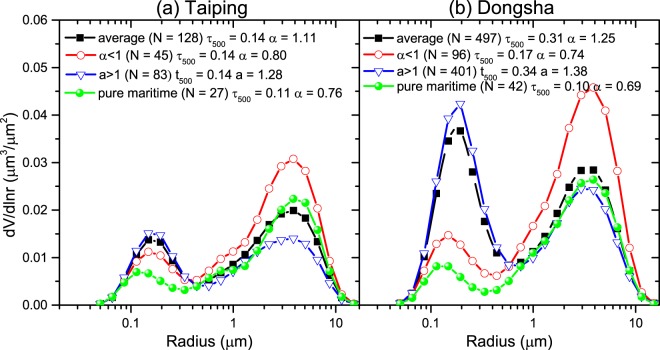
Average aerosol volume size distributions in the total column for Taiping(**a**) and Dongsha(**b**).

To retrieve the multi-source influence factors of “pure maritime aerosols”, the aerosol volume size distribution (VSD) is an essential parameter to discussed. As to the results of Smirnov *et al*. 2002 and 2003 based on the measurement in maritime and coastal areas global^10,11^, the “pure maritime aerosols” have the limitation of AOD (500 nm) smaller than 0.15 and AE less than 1. In Fig. 3, the pure maritime aerosol VSDs are the average of the data that meets the criteria mentioned above. We divided the whole inversion dataset (Level 2.0) to two class with the criterion of AE larger than 1 and less than 1 to character the particle size. In Fig. 3(a), the black line shows the average VSD in Taiping with relatively higher volume concentration of coarse-mode particles (V_*f*_ = 0.02 *μm*^3^/*μm*^2^, V_*c*_ = 0.04*μm*^3^/*μm*^2^). By comparing two datasets with different AE range (red and blue line), the coarse mode volume concentration has the great variation but the fine mode changes little, which demonstrates  that the coarse-mode particles are dominated in the change of AE in Taiping. The green line shows the pure maritime that the fine-mode volume concentration of pure maritime aerosol is obviously less than the averaging value of other datasets.

In Fig. 3(b), the average VSD has the larger fine-mode volume concentration than the coarse-mode one. Moreover, in Dongsha the volume concentrations in fine and coarse mode are obviously larger than that of Taiping in the two datasets bounded by AE is equal to 1, respectively. Compared with the pure maritime VSD, the fine- and coarse-mode volume concentrations in Dongsha have  a large scope variety, that is, the complex sources with the small or large size particles have effects on the Dongsha site. As shown in Fig. 3, the values of N in the legend are the amounts of the data involved in averaging. In Taiping and Dongsha, the pure maritime aerosol has a proportion of 20% and 8%, respectively. Moreover, the dataset dominated by the small particles (AE > 1) plays an leading role in both two sites. Without considering other aspects, the background in the study area mainly consists of sea spray or clean continental and rural background. But in the analysis of VSD in the two sites, it is likely that the background aerosol over the South China sea has been influenced by the anthropogenic aerosol.

### Backward air trajectory

**Figure 4:**
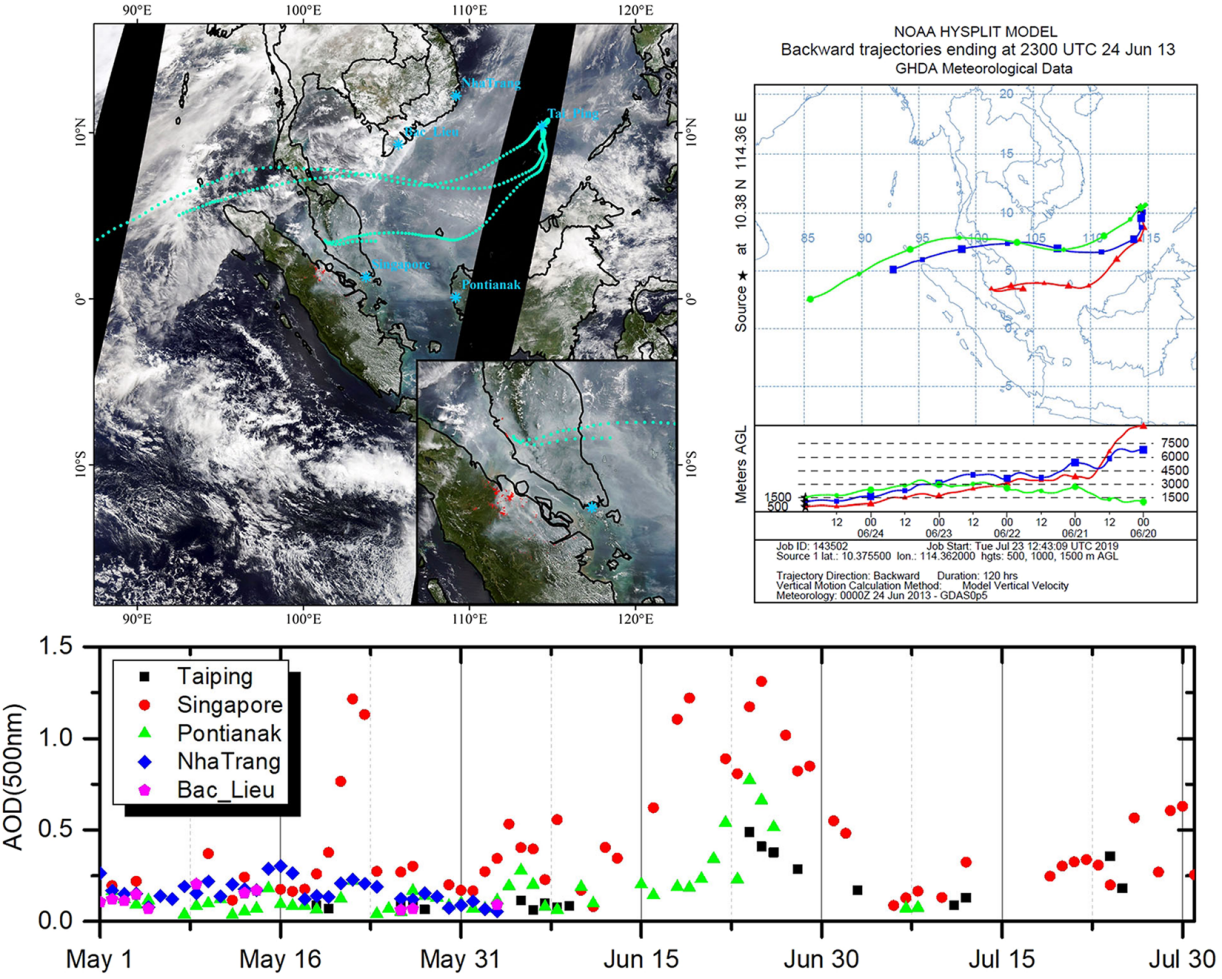
The backward trajectory from Taiping Island on 24^th^ Jun, 2013 and the time series of AODs(500 nm) in the sites around the backward trajectory. The satellite imagery is from the NASA Worldview application (https://worldview.earthdata.nasa.gov/) of NASA’s Earth Observing System Data and Information System (EOSDIS) and the red pattern spots are from the Terra and Aqua combined MCD64A1 Version 6 burned area data product, which were produced using ArcGIS (version 10.2).

Information on various meteorological and spatial fields can increase our understanding of the origins of different masses. During the measurement period, there are only 6 pieces of valid inversion data (Level 2.0) in Taiping site, which is from 24^th^ Jun to 26^th^,2013. As demonstrated in Fig. 4, Taiping Island was traced from Sumatra, located in the Maritime Continent. In the top left figure (the true color image), the red pattern indicates the burned area from the MODIS MCD64A1 Version 6 Burned Area data product, which is combined by Terra and Aqua, a monthly global gridded 500 meters product. Trans-boundary haze events in South Asia are associated with large forest and peatland fires in Indonesia. Our investigation of the June 2013 fires in Sumatra that burning an estimated 163,336 ha, including 137,044 ha (84%) on peat, and most burning was confined to deforested lands (82%; 133,216 ha)^21^. The bottom figure shows the time series of AODs at 500 nm. As expected, there is a increasing pattern from 15^th^ to 30^th^. The sites of Pontianak and Singapore were most seriously affected.

**Figure 5:**
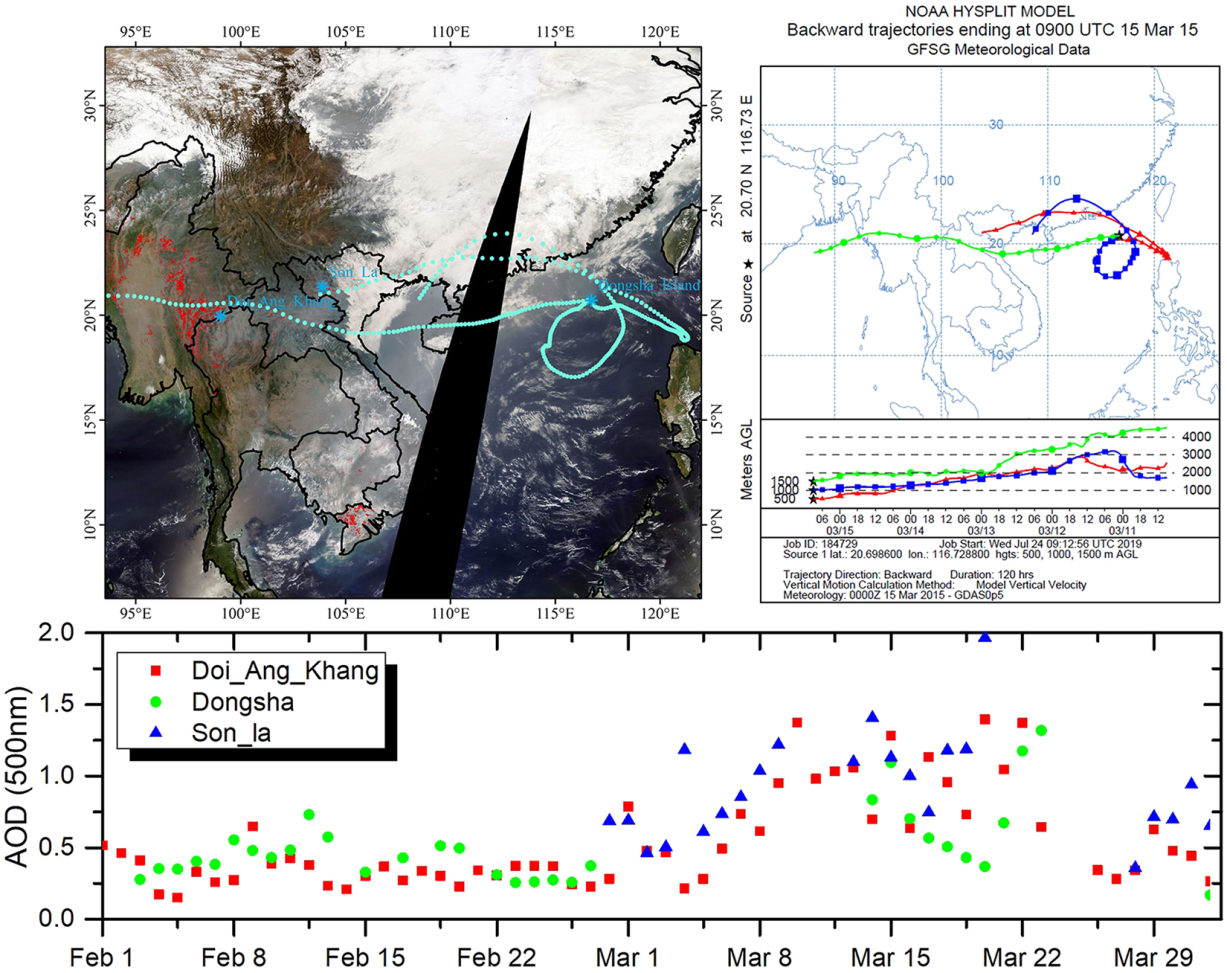
The backward trajectory from Dongsha Island on 15^th^ Mar, 2015 and the time series of AODs(500 nm) in the sites around the backward trajectory. The satellite imagery is from the NASA Worldview application (https://worldview.earthdata.nasa.gov/) of NASA’s Earth Observing System Data and Information System (EOSDIS) and the red pattern spots are from the Terra and Aqua combined MCD64A1 Version 6 burned area data product, which were produced using ArcGIS (version 10.2).

The Indochina Peninsula is another area with large forest and peatland fires around the region of the South China Sea. In spring, fire is a deeply rooted agricultural instrument in Southeast Asia. Burning associated with agriculture and pasture as well as deforestation forestation burning, are the common seasonal features. Moreover, the emissions with the long-range transport events make a significant contribution to atmospheric and climate change^3,22^. In 15^th^ Mar, 2015, Dongsha Island was influenced by the fire agriculture in Indochina Peninsula. As shown in Fig. 5, the AODs of the sites around the backward trajectory have an increasing value when it comes to March.

**Figure 6:**
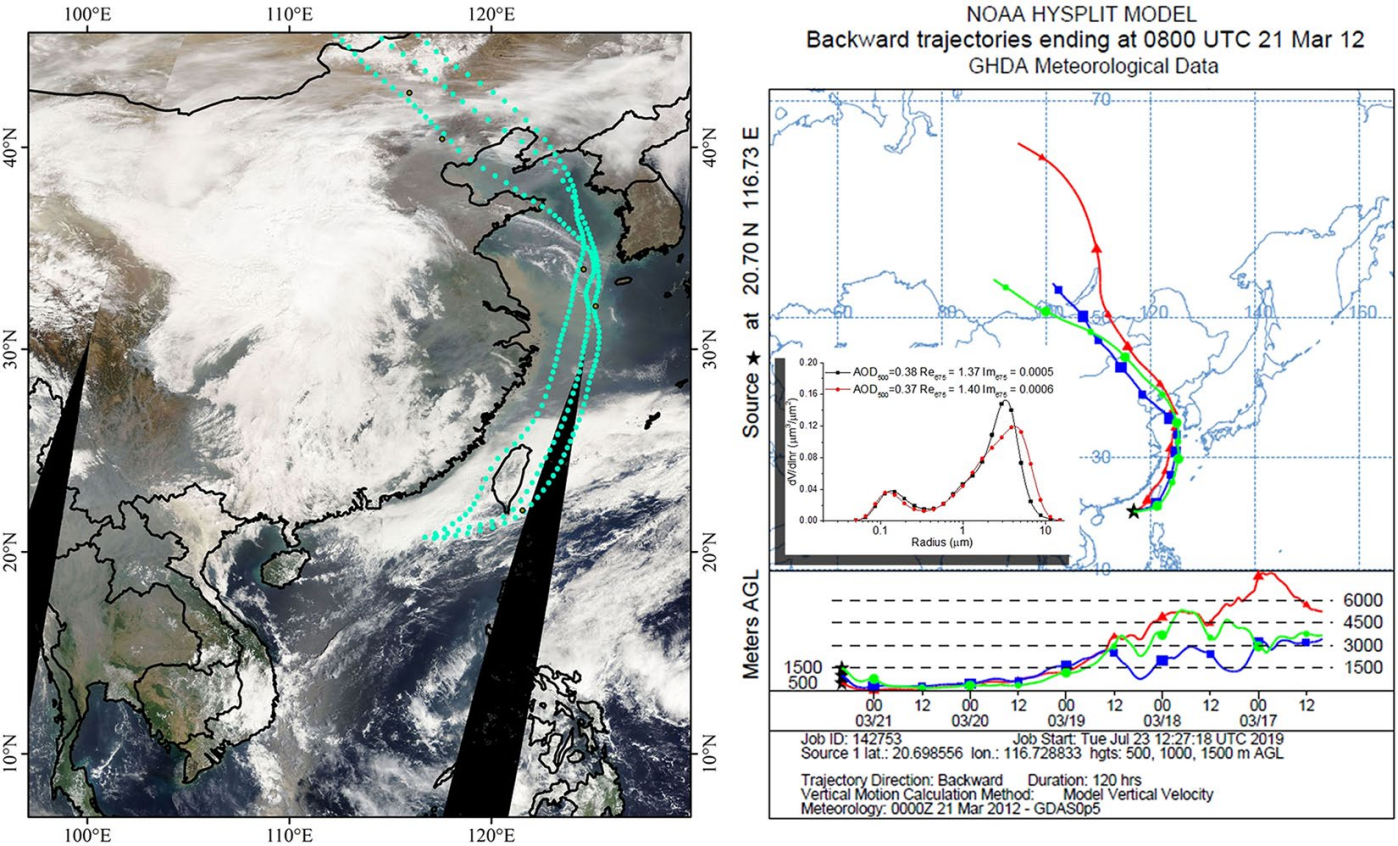
The backward trajectory from Dongsha Island on 21^th^ Mar, 2012. The satellite imagery is from the NASA Worldview application (https://worldview.earthdata.nasa.gov/) of NASA’s Earth Observing System Data and Information System (EOSDIS), which was produced using ArcGIS (version 10.2).

In spring, the dust from the northern China and Mongolia would influence on a large space range. In Fig. 3, the coarse-mode volume concentration has the unusually high values in Dongsha Island. In Fig. 6, the trajectories represent longest path, originating from elevated region over northern China and Mongolia and passing through the coastal areas of China. In addition, the VSDs shown in Fig. 6 have an obviously large coarse-mode volume concentrations. The similar result was also report in many studies^7–9^.

Furthermore, Dongsha Island is also influenced by West Pacific and Philippines^6^. Comparing the analysis of both sites, Dongsha Island has more complicated aerosol component due to the various sources and Taiping Island is relatively cleaner. Nevertheless, the properties of two sites are more complex and special in comparison with the other sites of island (Atlantic Ocean, Pacific Ocean, Indian Ocean) as illustrated in Smirnov^11^.

## Discussion

As mentioned above, the aerosol properties above the South China Sea vary considerably, depending on the anthropogenic source mainly. The region of the South China Sea has a monsoonal climate, which would be construed as a “wet” or “dry” season. The human element, including anthropogenic activities such as biomass burning co-vary with meteorology can be considered in the effect of monsoonal climate. So the seasonal maritime aerosol properties would be discussed in this section.

As to Level 2.0 restriction, the complex refractive index (CRI) is inverted as AOD at 440 nm more than 0.4 but VSD has no threshold. So we could not get the maritime aerosol models over the South China Sea due to the lower AOD value over ocean. If the Level 2.0 data are used for modeling, the results are lack of representativeness because of the little data.

So we chose Level 1.5 data after screening cloud. Dubovik *et al*. pointed out that the errors in retrieved refractive index of greater than 0.05 for the real part and 80–100% for the imaginary part should be expected as AOD at 440 nm is relatively low (less than 0.20)^19^. However, we decided to employ the average refractive index that simple averaging of all retrievals and all wavelengths. As illustrated in Table 1, the maritime aerosol models of Taiping and Dongsha in different seasons could represent the south and north of the South China Sea, respectively.

**Table 1:** The seasonal maritime aerosol models in Taiping and Dongsha, to represent the south and north of the South China Sea.

Season	Site	R_*f*_ (μm)	R_*c*_ (μm)	V_*f*_ (μm^3^/μm^2^)	V_*c*_ (*μm*^3^/*μm*^2^)	std_*f*_	std_*c*_	Re	Im
Spr	Taiping	0.16	2.55	0.01	0.03	0.46	0.72	1.505	0.006
	Dongsha	0.18	2.57	0.06	0.06	0.45	0.68	1.452	0.006
Sum	Taiping	0.18	2.52	0.03	0.06	0.44	0.71	1.493	0.004
	Dongsha	0.19	2.54	0.03	0.03	0.44	0.70	1.477	0.010
Aut	Taiping	0.18	2.68	0.02	0.05	0.44	0.69	1.497	0.004
	Dongsha	0.19	2.86	0.04	0.04	0.47	0.67	1.447	0.008
Win	Taiping	0.18	2.78	0.02	0.06	0.47	0.71	1.480	0.003
	Dongsha	0.20	2.88	0.05	0.06	0.50	0.63	1.445	0.003

*R*_*f*_ and *R*_*c*_ are median radii, *std*_*f*_ and *std*_*c*_ are standard deviations, *V*_*f*_ and *V*_*c*_ are volume concentrations. The subscripts f and c are fine and coarse modes. Re and Im are the real and imaginary parts of complex index, respectively.

**Figure 7:**
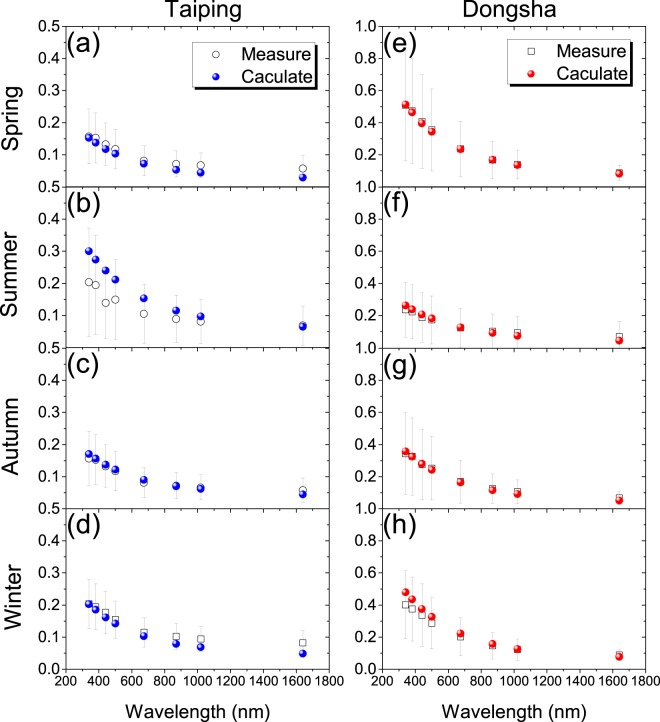
Comparison of spectral aerosol optical depths computed in the spectral range 340–1860 nm using the size distribution and average refractive index in Table 1 to the average aerosol optical depths measured in AERONET. Vertical error bars show plus or minus one standard deviation of the average measures value.

Figure 7 presents AODs computed in the spectral range 340–1860 nm using the average VSD and CRI in different seasons, compared with the average of the simultaneously measured AODs. All the subgraphs in Fig. 7 reflect general consistency among measured and calculated AODs. Although in Fig. 7(b,h), the calculated AODs bias high versus measured *τ*_*m*_, the difference between measured and computed AODs lies within one standard deviation. In Taiping, the bias value has a little increase as the increasing wavelength, which is consist with the characteristics of the larger coarse mode volume concentration that the large particles are more sensitive in the longer wavelengths.

Overall, the agreement between simulated and measured AODs is reasonable and accordingly we infer that the simply parameterized maritime aerosol models in the South China Sea, which can be employed to characterize the equivalent optical and microphysical properties in the northern and southern parts of the study area.

## Conclusion

In this study, we investigated the aerosol optical and microphysical properties of the South China Sea with the ground-based sites of AERONET (Taiping and Dongsha Island). Moreover, the complicated sources including how the origin co-vary with the maritime aerosol properties have been considered. In details, we draw the following conclusions:

1. In the comparison of other island sites, Taiping and Dongsha have the wide range of daily AOD (500 nm) and AE frequency distribution. In Taiping, the vast majority of AODs are less than 0.2, but that of Dongsha shows the wider distribution of AODs from 0 to 0.6. AE frequency distribution in Taiping peaks at the range of 0.75–1.25. However, in Dongsha, more data are in the range of AE larger than 1.
2. As to the microphysical properties, there is a variation in the coarse-mode volume concentration in Taiping Island but less change in the fine mode. But in Dongsha Island, as AOD increasing, it is equally likely that the fine- or coarse-mode volume concentration changes. It suggests the more complex aerosol components in Dongsha. Based on the backward air trajectory, the biomass burning is one main source of pollution. Moreover, the dust from North China and Mongolia also has an important effect on the coastal zone and island in the South China Sea.
3. The maritime aerosol models of Taiping and Dongsha in different seasons have been analyzed to represent the south and north of the South China Sea, respectively, which employed the seasonal average VSD as well as the average refractive index that simple averaging of all retrievals and all wavelengths.

## Supplementary information

Supplementary information
