# Supplementary material for: Maritime Aerosol Optical and Microphysical Properties in the South China Sea Under Multi-source Influence
Source: Sci Rep. 2019 Nov 28;9:17796. doi: 10.1038/s41598-019-54483-6 (PMC6882811; doi:10.1038/s41598-019-54483-6)
Supplement: Supplementary file 1 — Supplementary information [file 41598_2019_54483_MOESM1_ESM.docx]

# Maritime Aerosol Optical and Microphysical Properties in the South China Sea Under Multi-source Influence

Chi Zhang ^1, 2,*^, Zhengqiang Li ^1^, Hua Xu^1^, Yisong Xie ^1^,Donghui Li ^1^

^1^ State Environmental Protection Key Laboratory of Satellite Remote Sensing, Aerospace Information Research Institute, Chinese Academy of Sciences, Beijing 100101, China;

^2^ University of Chinese Academy of Sciences, Beijing 100049, China;

***** Correspondence: [zhangchi@radi.ac.cn](mailto:zhangchi@radi.ac.cn);

**Supplementary Information:**

**Supplementary Tables:**

**Supplementary Table S1.** The 22 bins of aerosol volume size distribution (VSD, ${{\mu m}^{3}}/{{\mu m}^{2}}$) of Taiping and Dongsha, which is the supplementary information of Figure 3.

| Radius  $\mu m$ | Taiping | | | | Dongsha | | | |
| --- | --- | --- | --- | --- | --- | --- | --- | --- |
|  | Average | AE>1 | AE<1 | Pure marine | Average | AE>1 | AE<1 | Pure marine |
| 0.05 | 0.000 | 0.000 | 0.000 | 0.000 | 0.000 | 0.000 | 0.000 | 0.000 |
| 0.07 | 0.002 | 0.002 | 0.002 | 0.002 | 0.002 | 0.003 | 0.002 | 0.002 |
| 0.09 | 0.006 | 0.006 | 0.005 | 0.005 | 0.010 | 0.011 | 0.007 | 0.005 |
| 0.11 | 0.011 | 0.011 | 0.009 | 0.007 | 0.024 | 0.026 | 0.013 | 0.008 |
| 0.15 | 0.014 | 0.015 | 0.011 | 0.007 | 0.035 | 0.039 | 0.015 | 0.008 |
| 0.19 | 0.013 | 0.015 | 0.010 | 0.005 | 0.037 | 0.042 | 0.013 | 0.006 |
| 0.26 | 0.009 | 0.010 | 0.008 | 0.004 | 0.028 | 0.033 | 0.009 | 0.004 |
| 0.33 | 0.006 | 0.006 | 0.005 | 0.003 | 0.018 | 0.020 | 0.007 | 0.003 |
| 0.44 | 0.004 | 0.004 | 0.005 | 0.004 | 0.011 | 0.012 | 0.006 | 0.003 |
| 0.58 | 0.005 | 0.004 | 0.007 | 0.006 | 0.008 | 0.008 | 0.008 | 0.005 |
| 0.76 | 0.007 | 0.005 | 0.010 | 0.007 | 0.009 | 0.008 | 0.012 | 0.008 |
| 0.99 | 0.009 | 0.007 | 0.011 | 0.007 | 0.011 | 0.010 | 0.017 | 0.011 |
| 1.30 | 0.011 | 0.009 | 0.013 | 0.008 | 0.014 | 0.013 | 0.021 | 0.014 |
| 1.71 | 0.014 | 0.011 | 0.018 | 0.011 | 0.019 | 0.017 | 0.028 | 0.017 |
| 2.24 | 0.017 | 0.013 | 0.025 | 0.016 | 0.025 | 0.022 | 0.037 | 0.022 |
| 2.94 | 0.019 | 0.014 | 0.029 | 0.020 | 0.028 | 0.025 | 0.044 | 0.026 |
| 3.86 | 0.020 | 0.014 | 0.031 | 0.022 | 0.028 | 0.024 | 0.046 | 0.026 |
| 5.06 | 0.018 | 0.013 | 0.028 | 0.022 | 0.024 | 0.020 | 0.041 | 0.024 |
| 6.64 | 0.013 | 0.010 | 0.020 | 0.016 | 0.016 | 0.013 | 0.028 | 0.016 |
| 8.71 | 0.006 | 0.005 | 0.009 | 0.007 | 0.007 | 0.006 | 0.012 | 0.007 |
| 11.43 | 0.002 | 0.001 | 0.002 | 0.002 | 0.002 | 0.002 | 0.003 | 0.002 |
| 15.00 | 0.000 | 0.000 | 0.000 | 0.000 | 0.000 | 0.000 | 0.000 | 0.000 |

**Supplementary Table S2.** The parameter of Taiping on 24^th^ and 25^th^ Jun, 2013, which is the supplementary information of Figure 4. $R_{f}$ and $R_{c}$ are median radii, ${std}_{f}$ and ${std}_{c}$ are standard deviations, $V_{f}$ and $V_{c}$ are volume concentrations. The subscripts f and c are fine and coarse mode. Re and Im are the real and imaginary part of complex index, respectively.

|  |  | Taiping | | | | | |
| --- | --- | --- | --- | --- | --- | --- | --- |
|  | Date | 2013-6-24 | 2013-6-25 | 2013-6-25 | 2013-6-25 | 2013-6-25 | 2013-6-26 |
|  | Time | 23:12:58 | 00:20:34 | 00:46:43 | 23:14:14 | 23:37:26 | 00:46:52 |
|  | AOD(440nm) | 0.50 | 0.48 | 0.48 | 0.44 | 0.43 | 0.42 |
| Single scattering Albedo | 440nm | 0.99 | 0.97 | 0.98 | 0.97 | 0.97 | 0.97 |
|  | 675 nm | 0.99 | 0.98 | 0.99 | 0.96 | 0.96 | 0.98 |
|  | 870 nm | 0.99 | 0.96 | 0.98 | 0.94 | 0.93 | 0.97 |
|  | 1020 nm | 0.99 | 0.94 | 0.97 | 0.94 | 0.92 | 0.96 |
| Complex refractive index  Real part | 440 nm | 1.413 | 1.512 | 1.509 | 1.515 | 1.506 | 1.492 |
|  | 675 nm | 1.460 | 1.521 | 1.515 | 1.494 | 1.490 | 1.486 |
|  | 870 nm | 1.477 | 1.528 | 1.522 | 1.489 | 1.488 | 1.489 |
|  | 1020 nm | 1.479 | 1.519 | 1.512 | 1.480 | 1.480 | 1.476 |
| Complex refractive index  Imaginary Part | 440 nm | 0.0006 | 0.0027 | 0.0018 | 0.0031 | 0.0034 | 0.0029 |
|  | 675 nm | 0.0005 | 0.0018 | 0.0010 | 0.0045 | 0.0044 | 0.0018 |
|  | 870 nm | 0.0005 | 0.0036 | 0.0016 | 0.0057 | 0.0065 | 0.0030 |
|  | 1020 nm | 0.0005 | 0.0050 | 0.0021 | 0.0060 | 0.0083 | 0.0040 |
| Volume size distribution | V_f_ | 0.06 | 0.04 | 0.04 | 0.04 | 0.04 | 0.04 |
|  | R_f_ | 0.16 | 0.19 | 0.19 | 0.21 | 0.21 | 0.20 |
|  | std_f_ | 0.44 | 0.39 | 0.40 | 0.39 | 0.38 | 0.40 |
|  | V_c_ | 0.08 | 0.09 | 0.07 | 0.06 | 0.06 | 0.06 |
|  | R_c_ | 1.75 | 1.92 | 1.67 | 1.80 | 1.77 | 1.68 |
|  | std_c_ | 0.71 | 0.75 | 0.67 | 0.73 | 0.71 | 0.67 |

**Supplementary Table S3.** The parameter of Dongsha on 15^th^ and 16^th^ Mar, 2015, which is the supplementary information of Figure 5.$R_{f}$ and $R_{c}$ are median radii, ${std}_{f}$ and ${std}_{c}$ are standard deviations, $V_{f}$ and $V_{c}$ are volume concentrations. The subscripts f and c are fine and coarse mode. Re and Im are the real and imaginary part of complex index, respectively.

|  | Date | 2015-3-15 | 2015-3-15 | 2015-3-15 |
| --- | --- | --- | --- | --- |
|  | Time | 01:05:23 | 08:57:03 | 23:51:05 |
|  | AOD(440nm) | 1.07 | 1.48 | 1.03 |
| Single scattering Albedo | 440nm | 0.94 | 0.93 | 0.92 |
|  | 675 nm | 0.95 | 0.91 | 0.93 |
|  | 870 nm | 0.94 | 0.88 | 0.91 |
|  | 1020 nm | 0.93 | 0.87 | 0.90 |
| Complex refractive index  Real part | 440 nm | 1.410 | 1.473 | 1.381 |
|  | 675 nm | 1.449 | 1.479 | 1.421 |
|  | 870 nm | 1.460 | 1.475 | 1.432 |
|  | 1020 nm | 1.463 | 1.469 | 1.435 |
| Complex refractive index  Imaginary Part | 440 nm | 0.0083 | 0.0125 | 0.0109 |
|  | 675 nm | 0.0065 | 0.0132 | 0.0084 |
|  | 870 nm | 0.0064 | 0.0142 | 0.0089 |
|  | 1020 nm | 0.0066 | 0.0146 | 0.0092 |
| Volume size distribution | V_f_ | 0.14 | 0.17 | 0.15 |
|  | R_f_ | 0.20 | 0.21 | 0.19 |
|  | std_f_ | 0.46 | 0.50 | 0.43 |
|  | V_c_ | 0.06 | 0.08 | 0.06 |
|  | R_c_ | 2.72 | 3.88 | 3.12 |
|  | std_c_ | 0.65 | 0.55 | 0.68 |

**Supplementary Table S4.** The parameter of Dongsha on 21^th^ Mar, 2012, which is the supplementary information of Figure 6. $R_{f}$ and $R_{c}$ are median radii, ${std}_{f}$ and ${std}_{c}$ are standard deviations, $V_{f}$ and $V_{c}$ are volume concentrations. The subscripts f and c are fine and coarse mode. Re and Im are the real and imaginary part of complex index, respectively.

|  |  | Dongsha | | |
| --- | --- | --- | --- | --- |
|  | Date | 2012-3-21 | 2012-3-21 | 2012-3-22 |
|  | Time | 23:22:45 | 23:44:11 | 00:56:25 |
|  | AOD(440nm) | 0.41 | 0.40 | 0.43 |
| Single scattering Albedo | 440nm | 0.98 | 0.98 | 0.99 |
|  | 675 nm | 0.99 | 0.99 | 0.99 |
|  | 870 nm | 0.99 | 0.98 | 0.99 |
|  | 1020 nm | 0.99 | 0.98 | 0.99 |
| Complex refractive index  Real part | 440 nm | 1.347 | 1.374 | 1.425 |
|  | 675 nm | 1.377 | 1.400 | 1.422 |
|  | 870 nm | 1.392 | 1.412 | 1.425 |
|  | 1020 nm | 1.394 | 1.412 | 1.421 |
| Complex refractive index  Imaginary Part | 440 nm | 0.0006 | 0.0006 | 0.0005 |
|  | 675 nm | 0.0005 | 0.0006 | 0.0005 |
|  | 870 nm | 0.0006 | 0.0011 | 0.0005 |
|  | 1020 nm | 0.0005 | 0.0013 | 0.0005 |
| Volume size distribution | V_f_ | 0.05 | 0.04 | 0.03 |
|  | R_f_ | 0.16 | 0.15 | 0.18 |
|  | std_f_ | 0.48 | 0.49 | 0.52 |
|  | V_c_ | 0.20 | 0.21 | 0.23 |
|  | R_c_ | 2.40 | 2.68 | 2.81 |
|  | std_c_ | 0.64 | 0.71 | 0.70 |
